# Outcomes of an integrated practice unit for vulnerable emergency department patients

**DOI:** 10.1186/s12913-023-10067-9

**Published:** 2023-12-21

**Authors:** Deepa Borde, Denny Fe G. Agana-Norman, Robert Leverence, Lorrie Photos, Jon Shuster, Kiran Lukose, Jacqueline Pinkney, Joy Wright, Lori Waxenberg, Brandon Allen, Nila S. Radhakrishnan

**Affiliations:** 1 University of Florida College of Medicine, 1600 SW Archer Rd, Gainesville, FL 32608 USA; 2 University of Texas Medical Branch, 301 University Blvd, Galveston, TX 77555 USA; 3 University of Texas Health, 703 Floyd Curl Dr, San Antonio, TX 78229 USA; 4 University of Florida Health, 1600 SW Archer Rd, Gainesville, FL 32608 USA

**Keywords:** Super-utilizer patients, Multi-visit patients, Interdisciplinary care, Emergency department visits, Integrated practice unit, Healthcare utilization, Uninsured patients

## Abstract

**Background:**

An integrated practice unit (IPU) that provides a multidisciplinary approach to patient care, typically involving a primary care provider, registered nurse, social worker, and pharmacist has been shown to reduce healthcare utilization among high-cost super-utilizer (SU) patients or multi-visit patients (MVP). However, less is known about differences in the impact of these interventions on insured vs. uninsured SU patients and super high frequency SUs ($\ge$8 ED visits per 6 months) vs. high frequency SUs (4–7 ED visits per 6 months).

**Methods:**

We assessed the percent reduction in ED visits, ED cost, hospitalizations, hospital days, and hospitalization costs following implementation of an IPU for SUs located in an academic tertiary care facility. We compared outcomes for publicly insured with uninsured patients, and super high frequency SUs with high frequency SUs 6 months before vs. 6 months after enrollment in the IPU.

**Results:**

There was an overall 25% reduction in hospitalizations (p < 0.001), and 23% reduction in hospital days (p = 0.0045), when comparing 6 months before vs. 6 months after enrollment in the program. There was a 26% reduction in average total direct hospitalization costs per patient (p = 0.002). Further analysis revealed a greater reduction in health care utilization for uninsured SU patients compared with publicly insured patients. The program reduced hospitalizations for super high frequency SUs. However, there was no statistically significant impact on overall health care utilization of super high frequency SUs when compared with high frequency SUs.

**Conclusions:**

Our study supports existing evidence that dedicated IPUs for SUs can achieve significant reductions in acute care utilization, particularly for uninsured and high frequency SU patients.

**Trial Registration:**

IRB201500212. Retrospectively registered.

## Background

An analysis by the Agency for Healthcare Research and Quality (AHRQ) in 2012 showed that the sickest 5% of U.S. patients account for 58.9% of U.S. health care costs, and these figures have been relatively constant since the 1970s [1, 2]. Many of these high cost patients are super-utilizers (SUs), defined by the Robert Wood Johnson Foundation as, “individuals whose complex physical, behavioral, and social needs are not well met through the current fragmented health care system.” [3]. Multiple criteria for identifying super-utilizers exist, but no standard methodology is available for determining which criteria should be used for a specific population. Cluster analysis can aid in selecting characteristics from the literature that systematically differentiate super-utilizer groups from other beneficiaries [4, 5]. In 2013, several promising SU programs convened to outline key elements of success. While all interventions utilized a care team approach that typically consisted of a social worker, nurse, and community health worker, notably, successful interventions also implemented a patient-centered approach and emphasized a solid investment in human relationships [5, 6].

Robust integrated practice units (IPUs) have shown promise in reducing healthcare utilization by vulnerable patients. IPUs provide a multidisciplinary approach to patient care, typically involving a primary care provider, registered nurse, social worker, and pharmacist. Althaus and colleagues conducted a systematic review of primarily case management-based interventions aiming to reduce ED visits. Seven of the 11 reviewed studies showed significant reductions in ED utilization after intervention [7].

Several similar interventions that used IPUs have also shown marked reductions in acute care usage and costs [8, 9]. However, there are no published studies to our knowledge that have compared the impact of interdisciplinary programs on utilization outcomes with respect to the analysis of the patient’s payor status and frequency of ED utilization. The purpose of this study was to determine if establishing an IPU for our SU patient population would lead to decreased acute care utilization and decreased healthcare costs. In addition, we hoped to determine if frequency of ED use and insurance status would identify patient populations most likely to benefit from this intervention.

## Methods

### Study setting

This study was conducted at a southeastern US academic tertiary care facility housing a state-designated Level I Trauma center. With 946 licensed beds, this health system has on average 42,000 admissions and 100,000 adult ED visits per year. Our facility received grant funding from Centers for Medicare and Medicaid Services through the $35 Million Low Income Pool Award in the amount of $660,000 over 2 years.

### Selection of participants

According to Locker and colleagues, ED visits of more than 4 per year are considered to be nonrandom events, and thus suggested this as a cutoff for defining patients who are ED super-utilizers [10]. In the year prior to the opening of our IPU (November 1, 2011 to October 31, 2012), we identified 2,485 patients who presented to our adult ED more than 4 times, accounting for 23% of ED visits and 20% of our total ED and hospitalization costs. Given the large size of this group and the small scale of our planned IPU intervention, we elected to focus on a smaller subset of patients with greater than or equal to 4 ED visits in 6 months. This group was further divided into high frequency patients (4–7 visits in 6 months) and super high frequency patients ($\ge$ 8 visits in 6 months). During the study period of May 2012 to April 2015, a SU patient list was generated every 2 months on a rolling basis and was uploaded into our electronic medical record (EMR) so we could determine in real time, which SU patients were in the ED or hospitalized. The clinic Social Worker then contacted inpatient case managers recommending a referral to the clinic if appropriate. Enrollment in the IPU was optional, and those that attended the first IPU appointment were considered study participants.

### Intervention

The clinic was open five half days per week and staffed by 1 hospitalist-at any time, a social worker, a clinical pharmacist, a registered nurse, and an addiction and pain management psychiatrist. The clinic provided patients with intensive but temporary services with an eventual plan to transition to a medical home [11]. At the first visit, a social worker performed a needs assessment to identify barriers to care such as lack of insurance, housing, and transportation. This assessment also included screenings for substance abuse, depression, and low health literacy. On subsequent visits the social worker connected patients to several resources available in the community, health insurance, transportation vouchers, and medication assistance. Hospitalists who staffed the clinic provided limited primary care services, subspecialty referrals, and referrals to an embedded pain management and addiction psychiatrist. The pharmacist reconciled medications, provided education regarding medication compliance, put medication in pill boxes, monitored opioid use when indicated, and recommended affordable medication options. The specifics of these interventions are detailed elsewhere [5]. The clinic served a dual purpose as a post-discharge clinic for non-super utilizing uninsured patients; however, outcomes for these patients are not included in this study.

### Data

A retrospective chart review was conducted for the period of November 1, 2012 to April 30, 2015. All hospital utilization and demographic data were extracted from the EMR and cost data was extracted from the hospital data warehouse. We used a pre/post design such that each patient served as his/her own historical control comparing outcomes 6 months before vs. 6 months after the initial IPU appointment.

### Outcomes

Primary outcomes evaluated pre and post enrollment were ED visits, ED total direct costs, hospitalizations, hospital days, and hospitalization total direct costs. Attendance at the first clinic appointment was considered enrollment in the study and the starting point of the post enrollment period. Patients who died during the study period were excluded from the analysis, as their inclusion could artificially affect the outcomes measured.

### Analysis

For the enrolled SU patients, we compared the six-month period before vs. the first six-month period after enrollment in the clinic. Thus, each subject had two values for an outcome, be it number of visits or cost, denoted by Y_1_ (pre) and Y_2_ (post). This comparison is completely distribution-free but is conditional to the population who joined the SU program. The methods, following classical survey sampling ratio estimation, were selected prior to looking at any study data.

From central limit theory and the delta method, [12] using natural logarithms,

Log($\overline{Y}$_2_ /$\overline{Y}$_1_) has an asymptotic normal distribution with mean log(µ_2_ / µ_1_) and variance.

V={(σ_1_/ µ_1_)^2^+(σ_2_/ µ_2_)^2^ -2ρ[σ_1_ σ_2_ /(µ_1_ µ_2_)}/N.

where µ_j_ and σ_j_ (j = 1 and 2) are the population mean and population standard deviation of Y_1_ and Y_2,_ ρ is the correlation between Y_1_ and Y_2_ and N = sample size.

V can be replaced by, $\hat {V}$, where the sample moments (mean, standard deviation, and correlation replace the population quantity without changing the large sample distribution.

- The point estimate of µ_2_ / µ_1_ is $\overline{Y}$_2_ /$\overline{Y}$_1_.
- A 95% confidence interval for µ_2_ / µ_1_ is bounded by.

$\overline{Y}$_2_ /$\overline{Y}$_1_ exp(± 1.96sqrt[$\hat {V}$]) where exp() is the natural antilog.

Estimated_Pct_reduction = 100 [($$\overline{Y}$$_2_ /$$\overline{Y}$$_1_)-1]

Endpoints of the confidence limits are likewise calculated for percentage reduction.

The two-sided P-value is obtained from Z=|Log($\overline{Y}$_2_ /$\overline{Y}$_1_)|/Sqrt($\hat {V}$)and we compared this against the standard normal distribution.

## Results

### Characteristics of study subjects

The study population included 186 SU patients who had 4 or more ED visits in the 6 months prior to their first clinic appointment. Demographic information can be found in Table 1. Pre-enrollment indices can be found in Table 2. 93% of patients enrolled had a mental health diagnosis and 66% had a substance use diagnosis.

**Table 1:** Demographics of Patient Population

	N = 186
**Demographics**	No. (%)
SEX	
Female	105 (56)
Male	81 (43)
MARITAL STATUS	
Divorced	35 (19)
Life Partner/Significant Other	3 (2)
Married	32 (17)
Separated	12 (6)
Single	96 (52)
Widowed	8 (4)
RACE	
Black	81 (44)
Asian and Other	6 (4)
White	99 (53)
Ethnicity	
Not Hispanic	183 (> 95)
County	
Alachua County	135 (73)
Inside Catchment County (excluding Alachua)	45 (> 20)
Outside Catchment County	*
Payor Mix	
Blue Cross Blue Shield and Commercial/Managed Care	6 (3)
Medicaid	79 (42)
Medicare	31 (17)
Self-Pay (Uninsured)	70 (38)
Age at enrollment *	43.97 ± 12.72 SD
Pre-Enrollment Indices**	
All Patients (n = 186)	
ED Visits	4.54
ED Cost	$1,246
Hospitalizations	3.01
Hospital Days	13.80
Hospitalization Cost	$14,082.00
Mental Illness Diagnosis	93%
Substance Abuse Diagnosis	66%
Uninsured (n = 70)	
ED Visits	4.31
ED Cost	$1,134
Hospitalizations	2.10
Hospital Days	8.50
Hospitalization Cost	$9,560.00
Mental Illness Diagnosis	94%
Substance Abuse Diagnosis	63%
Publicly Insured (n = 110)	
ED Visits	4.55
ED Cost	$1,288
Hospitalizations	3.55
Hospital Days	15.83
Hospitalization Cost	$15419.00
Mental Illness Diagnosis	93%
Substance Abuse Diagnosis	68%
High ED Frequency (n = 135)	
ED Visits	2.86
ED Cost	$787.53
Hospitalizations	2.45
Hospital Days	12.85
Hospitalization Cost	$13,492.00
Mental Illness Diagnosis	92%
Substance Abuse Diagnosis	64%
Super High ED Frequency (n = 51)	
ED Visits	8.98
ED Cost	$2,460.00
Hospitalizations	4.49
Hospital Days	16.29
Hospitalization Cost	$15,643.00
Mental Illness Diagnosis	96%
Substance Abuse Diagnosis	71%

*Denotes mean ± standard deviation

**Denotes average

SOURCE: Authors’ analysis of patient population. Data was collected from the patients’ electronic medical record.

**Table 2:** Pre-enrollment indices of patients

	All Patients (n = 186)	Uninsured (n = 70)	Publicly Insured* (n = 110)	High ED Frequency (n = 135)	Super High ED Frequency(n = 51)
ED Visits	4.54	4.31	4.55	2.86	8.98
ED Cost	$1246.00	$1134.00	$1288.00	$787.53	$2460.00
Hospitalizations	3.01	2.1	3.55	2.45	4.49
Hospital Days	13.8	8.5	15.83	12.85	16.29
Hospitalization Cost	$14082.00	$9560.00	$15419.00	$13492.00	$15643.00
Mental Illness Diagnosis	93%	94%	93%	92%	96%
Substance Abuse Diagnosis	66%	63%	68%	64%	71

SOURCE: Authors’ analysis of patient population. Data was collected from the patients’ electronic medical record. *Data for 6 patients who were privately insured are not included in this analysis.

### Main results

Statistical analyses of all patients and 4 subgroups are summarized in Table 3. There was an 11% reduction in ED visits (p = 0.22), a 25% reduction in hospitalizations (p < 0.001), and a 23% reduction in hospital days (p = 0.0045), when comparing 6 months before versus 6 months after enrollment in the clinic for all patients enrolled in the study. There was also a 26% reduction in average total direct hospitalization costs per patient (p = 0.002).

Additional analysis showed that the intervention had a greater impact on uninsured patients compared to publicly insured patients (Table 3). For the uninsured subgroup, there was a 28% reduction in ED visits (p = 0.02), 29% reduction in ED costs (p = 0.028), 49% reduction in hospitalizations (p < 0.001), 50% reduction in hospitalization cost (p = 0.002), and a 44% reduction in hospital days (p = 0.005). In comparison, the only marginally significant outcome for the publicly insured subgroup was a 15% reduction in hospitalizations (p = 0.04).

**Table 3:** Analysis of estimated percent reduction before and after first Care One clinic visit

ALL PATIENTS (n = 186)	Relative Risk Reduction in % (95% CI)	P-Value
Emergency Department (ED) Visits	11 (0.00–26.19)	0.22
ED Cost	10 (0.00–25.48)	0.29
Hospitalizations	25 (13.99–35.25)	< 0.01
Hospital Days	23 (7.83–35.93)	< 0.01
Hospitalization Cost	26 (10.02–38.59)	< 0.01
**SELF-PAY (UNINSURED) (n = 70)**		
Emergency Department (ED) Visits	28 (3.33–46.58)	0.02
ED Cost	29 (3.81–46.85)	0.02
Hospitalizations	49 (33.88–60.67)	< 0.01
Hospital Days	44 (15.78–62.36)	< 0.01
Hospitalization Cost	50 (17.85–69.14)	< 0.01
**MEDICARE/MEDICAID (PUBLICLY INSURED) (n = 110)**		
Emergency Department (ED) Visits	-4 (0.00–17.04)	0.74
ED Cost	-4 (0.00–17.77)	0.72
Hospitalizations	15 (0.55–27.12)	0.04
Hospital Days	14 (0.00–27.45)	0.10
Hospitalization Cost	11 (0.00–25.31)	0.16
**5–7 VISITS (HIGH ED FREQUENCY) (n = 135)**		
Emergency Department (ED) Visits	27 (7.15–42.50)	0.01
ED Cost	24 (1.24–41.44)	0.03
Hospitalizations	28 (14.15–39.78)	< 0.01
Hospital Days	30 (11.35–44.23)	< 0.01
Hospitalization Cost	30 (10.47–45.97)	< 0.01
**8 OR MORE VISITS (SUPER HIGH ED FREQUENCY) (n = 51)**		
Emergency Department (ED) Visits	-2 (0.00–20.57)	0.85
ED Cost	-2 (0.00–21.01)	0.86
Hospitalizations	21 (0.79–37.73)	0.04
Hospital Days	10 (0.00–30.57)	0.45
Hospitalization Cost	15 (0.00–34.10)	0.22

SOURCE: Authors’ analysis of patient population. Data was collected from the patients’ electronic medical record.

Further subgroup analysis was conducted comparing high frequency (4–7 ED visits in 6 months) with super high frequency ($\ge$8 ED visits in 6 months) groups. In the high frequency subgroup, there was a 27% reduction in average ED visits (p = 0.01), 24% reduction in average ED cost (p-value= 0.039), 28% reduction in average hospitalizations (p < 0.001), 30% reduction in hospitalization cost (p = 0.005), and a 30% reduction in average hospital days (p = 0.003). In comparison, the super high frequency group outcomes were not statistically significant, with the exception of a 21% reduction in average hospitalizations (p = 0.04).

## Discussion

Our study supports existing evidence that a dedicated IPU for super-utilizers can achieve significant reductions in acute care utilization and costs. Our subgroup analysis additionally revealed a significant reduction in hospitalizations (p < 0.01) and costs (p < 0.01) for uninsured patients and high ED frequency patients with minimal reductions in utilization and costs in the publicly insured and super-high ED frequency subgroups.

These findings help identify the patient subsets that are most likely to benefit from targeted multidisciplinary interventions. The results of this study suggest that uninsured patients and patients with high ED frequency may benefit from embedding social services, pharmacist-led education, and chronic pain and addiction treatment into low or no cost primary care settings.

We initially suspected differences between high ED frequency and super-high ED frequency patient groups could be accounted for by differences in mental and substance use, however we found that 96% of patients in the super-high ED frequency group had a mental health diagnosis and 71% a substance use diagnosis, compared with 92% with mental health issue and 64% with substance use in the high ED frequency groups. The publicly insured and uninsured groups also lacked significant differences in frequency of mental health diagnosis and substance use. One potential explanation is that the diagnoses present in the medical record problem lists are not specific enough to distinguish between disabling diagnoses which require attention and diagnoses with less clinical severity which overall do not impact the patient’s outcomes.

Another possible explanation for the differences between these subgroups is that our uninsured and high ED frequency patients may not have been as medically ill as our insured and super high ED groups. Future study involving in depth chart review and inclusion of the Charlson Comorbidity Index may further help to determine if medical complexity was comparable in these groups.

This study is limited in that it was observational and was done at one center, making generalization difficult. It is based on comparisons between pre-enrollment and post-enrollment. There are potential biases in both directions. On the one hand, there could be selection bias in the eligibility to join the intervention program that could create regression to the mean. But on the other hand, the general health of these frequent users could be deteriorating, leading to increased utilization.

## Conclusion

In an era of acute care over-utilization, it is imperative that we develop targeted interventions that can ease preventable burdens on our healthcare system [13–15] High-utilizers with multiple chronic diseases and socioeconomic challenges often experience significant care fragmentation which can be exacerbated with each compounding condition [16]. The primary obstacle to delivering efficient supportive care lies in determining which individuals would benefit most from such targeted programs [5] Our study findings indicate that an IPU was associated with a potential reduction on acute care utilization and reduction in costs for uninsured and high ED frequency patients. Further study and investigation are needed to understand which components of the IPU may benefit uninsured and high ED frequency patients. In addition, future research should determine what types of interventions would better serve the publicly insured and super high frequency patient groups.

## Data Availability

The datasets generated and/or analysed during the current study are not publicly available due presence of patient health information and privacy concerns but are available from the corresponding author on reasonable request.
